# Reconstruction of Moderately and Severely Atrophic Scalp—A Multicentric Experience in Surgical Treatment of Patients Irradiated for Tinea Capitis in Childhood and Surgical Algorithm

**DOI:** 10.3390/medicina59091678

**Published:** 2023-09-17

**Authors:** Maja Nikolić Živanović, Milana Jurišić, Milana Marinković, Danica Grujičić, Aleksandar Stanimirović, Vuk Šćepanović, Mihailo Milićević, Nikola Jovićević, Goran Videnović, Vedrana Pavlović, Sanda Bogunović Stojičić, Milan Jovanović, Jelena Jeremić, Marko Jović, Rosanda Ilić, Milan Stojičić

**Affiliations:** 1 Clinic for Burns, Plastic and Reconstructive Surgery, University Clinical Center of Serbia, 11000 Belgrade, Serbia; 2 Faculty of Medicine, 11000 Belgrade, Serbia; 3 Clinic for Neurosurgery, University Clinical Center of Serbia, 11000 Belgrade, Serbia; 4 Department for Maxillofacial Surgery, Faculty of Medicine, University of Priština in Kosovska Mitrovica, 38220 Kosovska Mitrovica, Serbia; 5 Institute for Medical Statistics and Informatics, Faculty of Medicine, University of Belgrade, 11000 Belgrade, Serbia

**Keywords:** tinea capitis, favus, ringworm, radiation-induced meningioma, radiation-induced scalp defects, basal cell carcinoma, squamous cell carcinoma, aseptic bone necrosis, osteoradionecrosis, scalp reconstruction

## Abstract

*Background and Objectives*: Before the introduction of griseofluvin, the use of X-ray radiation was the treatment of choice for tinea capitis. More than half a century later various types of tumors have been found to be associated with childhood irradiation due to tinea capitis, most commonly cancers of the head and neck, as well as brain tumors. The often unusually aggressive and recurrent nature of these tumors necessitates the need for repeated surgeries, while the atrophic skin with an impaired vascular supply due to radiation often poses an additional challenge for defect reconstruction. We present our experience in the surgical treatment of such patients. *Materials and Methods*: This is a retrospective cohort study. In this study, 37 patients treated for acquired defects of the scalp with a history of irradiation therapy due to tinea capitis in childhood were included in this study, 24 male and 13 female patients. The mean age at the first appointment was 60.6 ± 7.8, with the youngest included patient being 46 and the oldest being 75 years old. Patients’ characteristics, surgical treatment, and complications were analyzed and a reconstructive algorithm was developed. *Results*: Local flaps were used for reconstruction in 34 patients, direct sutures were used in 10 patients and 20 patients received split-thickness skin grafts for coverage of both primary and secondary defects for reconstruction of flap donor sites. One regional flap and one dermal substitute covered by an autologous skin graft were also used for reconstruction. Complications occurred in 43.2% of patients and were significantly associated with the presence of comorbidities (*p* = 0.001), aseptic bone necrosis (*p* = 0.001), as well as skin atrophy in frontal, occipital, and parietal region (*p* = 0.001, *p* = 0.042 and *p* = 0.001, respectively). A significant correlation between major complications and moderate skin atrophy was found only in the parietal region (*p* = 0.026). *Conclusions*: Unfortunately, many protocols developed for scalp reconstruction are not applicable in the setting of severe or diffuse scalp skin atrophy associated with high tumor recurrence rate and radiation-induced vascular impairment, such as in tinea capitis patients in Serbia. An algorithm has been developed based on the authors’ experience in managing these patients.

## 1. Introduction

Reconstruction of scalp defects after oncologic surgery in plastic and reconstructive surgery still remains a challenge given the limited elasticity of the scalp soft tissue, with only defects 3 cm in diameter or smaller being able to be closed primarily [1,2]. The anatomy of galea and pericranium play a crucial role in the inelasticity of the scalp. Depending on the underlying pathology of the scalp defect, exact localization on the scalp, risk of tumor recurrence, tumor management in terms of previous surgeries or radiotherapy, the subsequent tissue fibrosis or atrophy, vascular compromise, as well as patient’s age, and comorbidities, the range of methods of choice for scalp defect reconstruction narrows.

During the 1950s, tinea capitis, also known as ringworm, herpes tonsurans, or favus, was one of the most common fungal follicle infections, most commonly on the scalp, caused by dermatophytic fungi, with the actual incidence at the time still unknown due to its underreporting. Reports of 500,000 children from North Africa and nearly 20,000 Israeli children are found reported in the literature with the disease spreading to Europe and the United States as well as former Yugoslavia [3]. Serbia is found to have one of the highest numbers of screened and treated children in Europe and North America (878,659 and 49,389 children, respectively) [4]. Before the introduction of griseofluvin, an antimycotic able to penetrate the hair follicle, the use of X-ray radiation (also known as the Kienboch–Adamson method) to remove hair followed by the application of topical antimycotics was the treatment of choice for tinea capitis [5,6]. The x-ray irradiation was found to be an effective way of epilation following 2–3 weeks of exposure. After shaving, the scalp area was divided into sections, each irradiated on one of 3–5 consecutive days during the 2–3-week period [7,8]. Zinc ointment was applied 1–2 days after irradiation. Even though exact protocols and doses of radiation received in Serbia are hard to obtain from the literature, a historic review including a recollection of one of the physicians reports the maximum doses given for tinea capitis to be 400 roentgens, an equivalent of 3,5 Gy. Most importantly, in the same historic review, at least four senior physicians were interviewed with all of them testifying their personal belief that the campaign, supported by UNICEF, was of great national public health importance at the time [3].

More than half a century later, the consequences of radiation therapy for tinea capitis have been emerging. Various types of tumors have been found associated with childhood irradiation due to ringworm infection, most commonly cancers of the head and neck, referring to basal cell carcinoma, squamous cell carcinoma of the skin as well as cancer of the lips, oral cavity, or pharynx, thyroid cancer, and brain tumors such as meningioma [9,10,11]. The often unusually aggressive and recurrent nature of these tumors necessitates the need for repeated surgeries, while the atrophic skin with an impaired vascular supply due to radiation often poses an additional challenge for defect reconstruction. A careful evaluation and planning of such surgeries is often necessary. Here, we present our experience in the surgical treatment of such patients, common complications, and management.

## 2. Materials and Methods

### 2.1. Study Design and Data Source

This is a retrospective cohort study. Patients treated for acquired defects of the scalp with a history of irradiation therapy due to tinea capitis in childhood were included in this study. All patients were treated by the corresponding and senior authors at either the Clinic for burns, plastic, and reconstructive surgery or the Clinic for neurosurgery, University Clinical Center of Serbia, depending on the underlying defect pathology, in the last 15 years. For all patients, preoperative, intraoperative, or postoperative photographs were available for interpretation. Patients with advanced disease, with a history of multiple previous surgeries not providing sufficient medical documentation regarding the treatment and course of the disease, were excluded.

After the approval of the Institutional Review Board, data were extracted from available medical records and photographs. Data included demographic data (age, sex, and residence), time of onset, tumor histopathology, history of tumor recurrence, as well as reconstruction method, complications, need for revisions, and patient comorbidities. Tumor types recorded included basal cell carcinoma (BCC), skin squamous cell carcinoma (sSCC), and meningioma. Other pathologies associated with irradiated tinea capitis patients and their frequencies included aseptic bone necrosis.

Comorbidities were grouped as cardiovascular, pulmonary, diabetes mellitus, or other. Cardiovascular comorbidities included hypertension, angina pectoris, myocardial infarction, cardiomyopathy, condition after coronary artery bypass grafting (CABG), arrhythmias, pulmonary thromboembolism, and heart failure. Pulmonary comorbidities included atelectasis, emphysema, chronic obstructive pulmonary disease, and asthma, while other comorbidities included breast cancer, hypothyroidism, and depression.

Reconstruction methods included primary closure, autologous skin grafting, local flap, regional flap, or dermal regeneration template, while complications included infection, wound dehiscence, skin graft loss, or flap necrosis and were classified as minor or major depending on the need for surgical revision.

Degree of skin atrophy was noted for the frontal, temporal, occipital, and parietal regions of the scalp in each patient and further classified as severe if skin thickness was 30% of normal skin, with loss of appendages and sudoriferous glands and apparent telangiectasias present, while moderate skin atrophy included skin thickness 60% of normal skin with any of the other signs absent. All patient photographs were carefully evaluated and compared to medical records.

### 2.2. Data Analysis

Descriptive statistics, including means and standard deviations for numerical variables and numbers and percentages for categorical variables, were used to characterize the study sample. Associations between categorical data were evaluated using the Pearson chi-square test or Fisher’s exact test. Student’s *t* test was used for numerical data to evaluate the differences between groups. In all analyses, the level of statistical significance was set at *p* ≤ 0.05. SPSS version 25 statistical software (Chicago, IL, USA) was used to perform the statistical analysis.

## 3. Results

### 3.1. Patient Characteristics

In this study, 37 patients matched the inclusion criteria. All patient characteristics are presented in Table 1. The mean age at the first appointment was 60.6 ± 7.8, with the youngest included patient being 46 and the oldest being 75 years old. There were no significant differences between genders regarding tumor types, reconstruction methods, or surgical complications.

### 3.2. Surgical Treatment and Complications

Local flaps were used for reconstruction in 34 patients, direct sutures were used in 10 patients, and 20 patients received split-thickness skin grafts for coverage of both primary (in 12 patients) and secondary defects (for flap donor sites in 8 patients). One regional flap and one dermal substitute (Integra^®^ regeneration template, Integra LifeSciences, Princeton, NJ, USA) covered by an autologous skin graft were also used for reconstruction. There were no significant differences regarding the chosen reconstructive method and the underlying pathology of the scalp defect.

Complications occurred in 43.2% of the patients, and were significantly associated with the presence of comorbidities (*p* = 0.001). Six major complications requiring surgical revisions were recorded. Partial flap necrosis occurred in 27.0% of patients, while only 2 events of wound dehiscence were noted. Partial skin graft loss occurred in 30.0% of skin transplants in both primary and secondary defects. The use of skin grafts as well as primary closure were significantly associated with tumor recurrence (*p* = 0.029 and *p* = 0.006, respectively). Infection, in terms of postoperative complications, occurred in 10.8% of patients. The occurrence of complications was not associated with any underlying pathology of defects nor a reconstructive method in particular, except for aseptic bone necrosis (*p* = 0.001).

Aseptic bone necrosis was more commonly found in patients with recurrent tumors (*p* = 0.029) as well as in patients with pulmonary comorbidities (*p* = 0.016). Characteristics of patients with aseptic bone necrosis and association with underlying defect pathology, reconstruction method, and complications are presented in Table 2.

The distribution and severity of skin atrophy are presented in Table 3. Differences in severity or localization of skin atrophy did not differ between genders. No correlation was found between skin atrophy localization or severity and reconstruction method, while in terms of complications, flap necrosis was significantly associated with all regions of severe atrophy (*p* = 0.052, *p* = 0.034, *p* = 0.006 and *p* = 0.021 for frontal, occipital, parietal, and temporal localization, respectively). Moderate skin atrophy was associated with flap necrosis only in the temporal region (*p* = 0.056). Major complications were associated with severe skin atrophy in frontal, occipital, and parietal region (*p* = 0.001, *p* = 0.042, and *p* = 0.001, respectively), while a significant correlation between major complications and moderate skin atrophy were found only in the parietal region (*p* = 0.026).

## 4. Discussion

**Figure 1:**
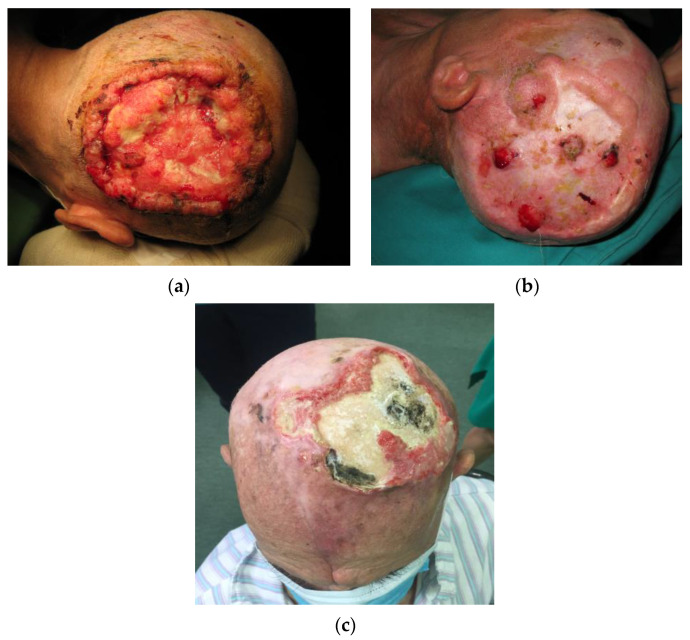
Clinical presentation of skin tumors in tinea capitis patients. (**a**) A locally destructive basocellular carcinoma (ulcus terebrans) with bone invasion. (**b**) A recurrent squamocellular carcinoma in a patient with multiple previous surgeries due to both recurrent basocellular and squamocellular carcinoma. (**c**) A neglected recurrent basocellular tumor with apparent bone invasion.

In our study, 86.5% of patients had skin cancers with BCC more commonly occurring than sSCC. 43.2% experienced a recurrence of the skin tumor, while 64.9% had multiple lesions. In a study exploring radiation-induced BCC, Zargari et al. found that 51.7% had recurrent lesions in the irradiated patient group compared to 10.3% of patients in the non-irradiated group, while 79.3% of patients had multiple lesions at the time of presentation [12]. More importantly, even though BCC is a slow-growing tumor in the general population, studies have found that in irradiated patients, 32.4% of BCC tumors were high-risk, aggressive tumors (Figure 1) [10].

Before deciding on the best reconstruction method for defects secondary to skin tumors in irradiated patients, localization, size of the tumor, risk of recurrence, depth, as well as underlying structures affected by the secondary defect, should be carefully evaluated. The goals of scalp reconstruction should be both functional and aesthetic. The principle of replacing “like with like” includes paying attention to skin quality, especially color matching and thickness. Maintaining an adequate hairline and reducing alopecia as well as scars with finely positioned incisions and attention to hair growth patterns are cosmetic factors related to successful scalp reconstruction [2,13]. Given the effects of radiation on the skin, as well as the high rate of tumor recurrence in these patients, selecting a strategy for reconstruction represents a significant challenge. Unfortunately, in this group of patients, the achievement of such goals is often impossible and the aim emphasizes obtaining adequate wound closure in these specific conditions. In general, the surgical treatment of scalp defects includes a wide range of procedures, from simple to complex: spontaneous healing—sanatio per secundam intentionem, closure by direct suturing, skin grafts of various thicknesses (partial to full thickness), a combination of dermal substitutes and skin grafts, local random or axial flaps (cutaneous and galeopericranial), regional flaps, use of tissue expanders in order to gain excess tissue or free tissue transfer [2,13].

**Figure 2:**
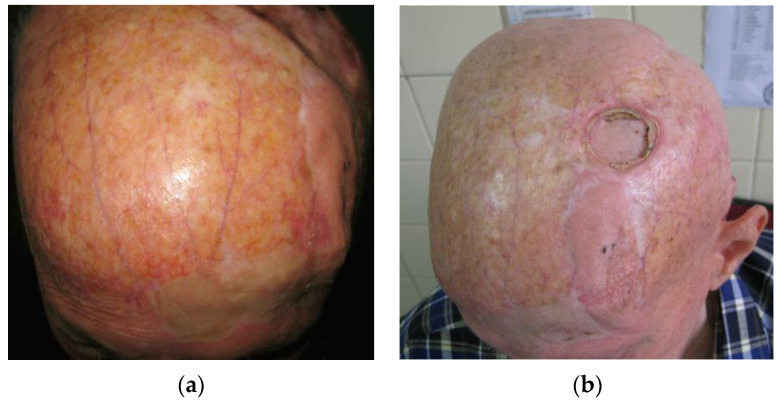
(**a**) Skin atrophy in tinea capitis patients. In this case, severe, diffuse skin atrophy with loss of skin appendages, hair follicles, sebaceous, and sudoriferous glands, accompanied by telangiectasia and blood vessel dilation is present. (**b**) The same, male patient presenting with aseptic necrosis. A frontal transpositional flap was previously used for the reconstruction of post-excisiona defect with clear differences in flap skin thickness and quality compared to the rest of the scalp. The flap was taken from a donor site of moderate skin atrophy.

Radiation-damaged skin, also known as chronic radiation dermatitis, usually presents as skin atrophy, loss of skin appendages, hair follicles, sebaceous, and sudoriferous glands, accompanied by additional radiation damage to blood vessels, insufficient oxygenation, and subsequent impaired healing. Pathophysiologic mechanisms behind radiation-caused skin damage suggest that the imbalance of proinflammatory and profibrotic cytokines as well as oxidative stress play a crucial role [14]. These changes can start after irradiation and last for months or even years. In these patients, the quality of the skin as well as vascular supply are impaired. Tissue fibrosis, reduction in the number of epidermal cells, thinning of various skin layers, as well as diminished natural healing mechanisms, subsequently render these patients difficult to manage, even after simple excisions (Figure 2) [15,16].

Direct closure is the simplest reconstructive technique, though sometimes difficult to perform on the scalp. If the mobility of the skin in the defect site is not limited by the scalp convexity, primary closure can usually be achieved in defects smaller than 3 cm in normal circumstances [13]. Tension-free reconstruction is always the goal and its benefits are reflected in the lower risk of skin edge necrosis and iatrogenic alopecia. In irradiated patients, skin atrophy and fibrosis are additional limiting factors in skin mobility and inelasticity, making even smaller defects a challenge for primary closure. In our study, direct suturing was used in 27.0% patients with wound dehiscence occurring in only one patient. There was no statistical difference between the sexes in the use of this approach. Furthermore, no significance was found between the use of this approach and the occurrence of complications at the surgical site. Even though it may seem like a simple and safe solution, risking radicality in tumor excision in primary closure is never acceptable, as demonstrated by a significantly greater rate of tumor recurrence in direct closures in our study (*p* = 0.006). Given the often more aggressive nature, skin tumors in these patients should always be considered as “high-risk”, with a tumor margin of >6 mm, often limiting the use of primary closure as the first-choice closure method [17,18].

**Figure 3:**
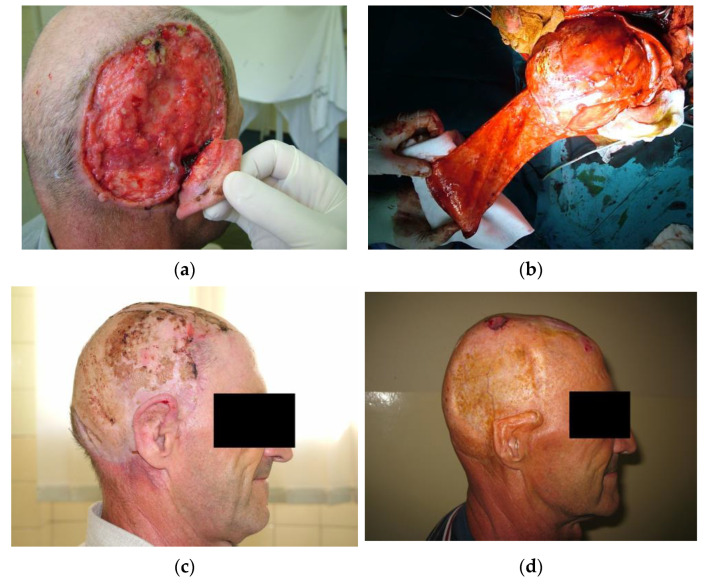
The use of transpositional flaps in scalp reconstruction. (**a**) A male patient presenting with ulcus terebrans with bone invasion. (**b**) After craniotomy, the defect was reconstructed with a transpositional flap while the donor site was covered by autologous skin grafts. (**c**) Four weeks postoperative. The flap survived without complications. Further adjuvant radiotherapy was indicated. (**d**) Seven years after surgery and adjuvant radiotherapy. The patient presented with a small recurrent basocellular carcinoma on the flap periphery. The post-excisional defect was reconstructed with a full-thickness autograft. The upper pole of the auricula sustained damage due to radiation therapy. The patient was not motivated for further reconstruction of the scalp or the auricula.

If tension-free closure by primary suture is impossible due to the inelasticity of the skin or a larger defect size, when possible, local skin flaps should be a method of choice. Local flaps are found to be appropriate for moderate-size scalp defects. Additionally, local flaps present the best solution in terms of “replacing like with like” [2,13,19,20]. Depending on the tumor type, size, and clinical behavior, the achievement of good aesthetic results should always be considered. An example of a careful flap design includes flaps located frontally where, if hair in these patients is present, the design should not allow the hairline, and most importantly, eyebrows to relocate. In addition, the incision should be performed underneath the hair follicle [13]. Good vascularization is crucial for flap survival. Additional factors influencing flap survival include proper flap base width, a lesser number of flaps, and fewer sutures in critical positions on the flap [19]. Additional factors contributing to reconstruction limitations include the presence of scar tissue often due to multiple previous surgeries as well as loss of scalp soft tissue volume in terms of atrophy. Local flaps should never be taken from a donor site of severe atrophy. Thus, the decision on flap selection and design is far more difficult compared to the reconstruction of the same size defects on healthy skin. In our study, local fasciocutaneous flaps were used for reconstruction in nearly 91.9% of patients (Figure 3).

Depending on the localization of the defect as well as the quality of the surrounding skin, transpositional, rotational, and interpolation flaps were most often used. Galeal scoring in order to increase flap length and reduce tension attributes to further vascular impairment and jeopardizes flap integrity. It is thus not recommended in atrophic skin. Partial necrosis occurred in 27.0% of patients, clinically presenting as epidermolysis of the flap, resolving without the need for surgical revision in most cases. Compared to the 0.7 to 5.5% incidence of flap necrosis on the scalp in the literature, flap necrosis occurs more commonly in irradiated patients due to impaired healing mechanisms and vascular supply [21]. Thus, using a conservative flap length or a wide-based flap in these patients is recommended. If a single flap could not be used to reconstruct a defect, transpositional flaps with autologous split-thickness skin grafts for donor site reconstruction should be considered superior to the use of multiple flaps in radiation-damaged skin. Major complications were found to be associated with severe skin atrophy in almost any region of the scalp (*p* = 0.001, *p* = 0.042, *p* = 0.001 for frontal, occipital, and parietal regions of severe skin atrophy, respectively) while moderate skin atrophy was significantly related to major complications in the parietal region (*p* = 0.026).

In the event of high tumor recurrence or other limitations for the use of local skin flaps, such as excessive scar tissue due to multiple previous surgeries, if the periosteum is intact, split- or full-thickness skin autografts can be used. The disadvantage of this approach is its poor aesthetic outcome due to differences in skin color and texture, contour irregularity, and a lack of skin adnexa, resulting in the impression of a patch. Autologous skin grafts were used in our study in 54.0% of defects with partial skin graft loss occurring in 30.0% of cases for both primary defect reconstruction as well as reconstruction of the local skin flap donor site. In our study, skin autografts as a method for primary, post-excisional defect reconstruction were mostly used in recurrent skin tumors as a last choice option.

**Figure 4:**
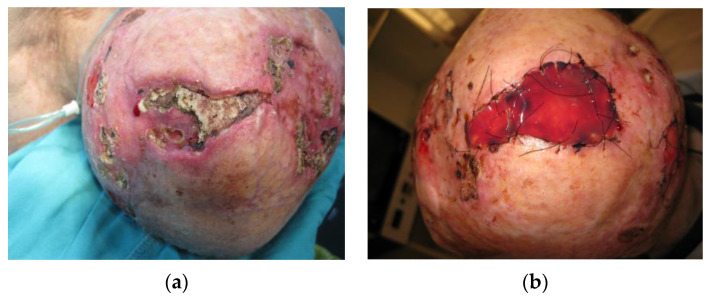
(**a**) A 74-year-old male patient with aseptic bone necrosis and recurrent basocellular tumor. In medical history, two previous myocardial infarctions, condition after CABG as well as chronic pulmonary obstructive disease were noted. The patient was receiving corticosteroid therapy at the moment of presentation. A severe diffuse atrophy of the scalp was present in all areas, except for the moderately atrophied frontal skin. (**b**) A full-thickness removal of the aseptic bone necrosis was performed, reaching dura, and a dermal regeneration template was used. After 3 weeks, the dermal regeneration template was covered with an autologous skin graft. Histopathological verification of aseptic bone necrosis in such cases is paramount before further reconstruction, differentiating from tumor bone invasion.

Avascular bone necrosis or aseptic bone necrosis is a condition most commonly found affecting the hip joint in the general population, caused by the impairment of bone tissue vascularization. Risk factors found to be associated with aseptic necrosis include corticosteroid therapy and excessive alcohol intake [22]. Cases of avascular bone necrosis in the head and neck are well documented and are commonly described as affecting the temporal bone. Additionally, radiation therapy has been found to be a significant risk factor [23]. Otherwise known as osteoradionecrosis, mechanisms behind this condition could be attributed to radiation-induced endothelial injury followed by decreased osteoblast proliferation together with increased osteoclast activity, in some cases. These changes accompanied by a decrease in the absolute number of osteocytes further lead to bone and fibrous connective tissue degeneration and death [24]. Studies exploring osteoradionecrosis of the temporal bone found bacterial colonization could also exacerbate such a condition [25]. Interestingly, in addition to spontaneous occurrence, aseptic necrosis most commonly appeared on flap donor sites reconstructed with split-thickness skin grafts. Given the vascular impairment as the main pathophysiological mechanism behind aseptic bone necrosis, this phenomenon could be attributed to additional trauma to the underlying bony structures caused by flap harvesting surgery. Surgical complications were significantly associated with aseptic bone necrosis (*p* < 0.001), primarily infection (*p* = 0.016), and partial autologous skin graft loss (*p* < 0.001). Pulmonary comorbidities were also correlated to the occurrence of aseptic bone necrosis (*p* = 0.016), possibly due to corticosteroid use in chronic obstructive pulmonary disease and/or inadequate oxygenation. Providing adequate vascularization in such a vascularly impaired region is the optimal goal, thus reconstruction with flaps, if applicable, presents a reasonable choice. An example of aseptic bone necrosis is shown in Figure 4.

Meningioma is a common intracranial neoplasm, classified by many histologic subtypes across 3 grades of malignancy. First-choice therapy for meningioma is a maximally safe resection for grade I tumors. Surgery plus optional or mandatory adjuvant radiotherapy for grade II and III tumors, respectively, are the treatments of choice, given the increased rate of recurrence even in the event of complete resection. Conversely, meningiomas are the most common secondary brain tumors following cranial radiotherapy, with a 9.5-fold increase in risk of meningioma occurrence in patients receiving a 1–2 Gy radiation dose in childhood [26]. Thus, adjuvant radiotherapy seems like an interesting paradox in the treatment of radiation-induced meningiomas (RIMs). RIMs are found to be more clinically aggressive than sporadic meningioma, as well as more likely to be multiple and reoccur after surgery [26].

**Figure 5:**
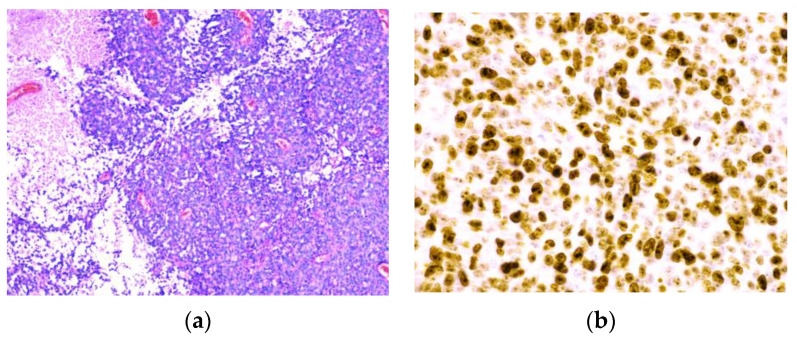
Pathohistological findings of anaplastic meningioma. (**a**) Anaplastic (malignant) menigioma resembling a carcinoma or a high-grade sarcoma. (**b**) A high Ki-67 proliferation index > 20.0%.

Anaplastic meningioma is the most commonly occurring histologic type of RIM, accounting for less than 5.0% of all meningiomas, with the worst prognosis (Figure 5) [27]. Radiation exposure has become one of the first theories regarding meningioma formation, along with early CNS trauma and hormone-induced/dependent meningioma formation [28]. The prognostic factors in patients with anaplastic meningioma are unclear and remain controversial. Several retrospective studies have reported the importance of gross total resection (GTR) and its association with better survival outcomes. Nonetheless, a consensus in the literature has been achieved regarding the importance of adjuvant radiation regardless of the extent of resection [29].

Given the aggressive nature of RIMs, one of the biggest challenges aside from RIM management remains defect reconstruction after resection in tinea capitis patients. After closure, protecting the calvarium from desiccation and infection by providing an adequate blood supply via vascularized tissue is one of the most important functional aspects. Surgery of recurrent or multiple meningiomas often includes large cranial defects, commonly reconstructed by the use of alloplastic implants such as titanium mesh, methyl methacrylate (MMA), hydroxyapatite, and polyetheretherketone (PEEK)—each with its strengths and limitations [30].

**Figure 6:**
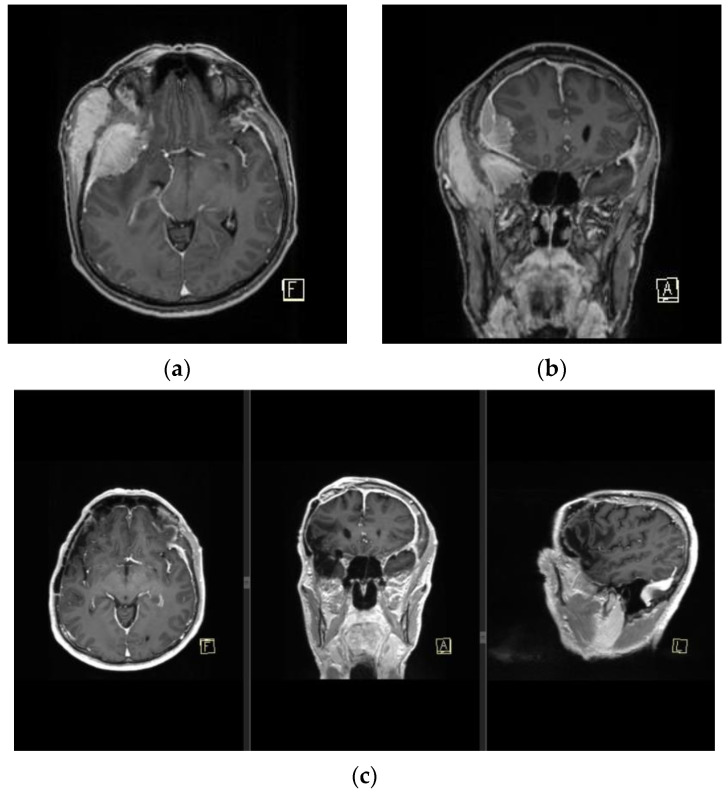
MRI finding of pan-meningiomatosis. (**a**,**b**) show preoperative MRI findings of an invasive anaplastic menigioma in a RIM patient due to tinea capitis radiation treatment in childhood, and prior to a wide Simpson gr I tumor resection with duroplasty and cranioplasty. (**c**) shows a postoperative MRI finding of the patient with a complete dural signal enhancement.

Depending on the meningioma surgical defect size, local, regional, or microvascular flaps can be implemented. Microvascular or free flaps are often the optimal solution when large defects or chronic infections are present, as well as when neurocranial structures or alloplastic materials are exposed [2,13,19,20]. Free flaps are able to provide adequate covering for implant hardware while also limiting donor site morbidity in an irradiation-impaired scalp skin quality [2,13,31]. The most common recipient blood vessels are the superficial temporal artery and vein. Blood vessels of the head and neck are commonly affected by radiation therapy, thus the choice of the recipient vessel should be carefully selected outside of the radiation segments and adequate blood supply should be verified by ultrasonography. The disadvantage of this method includes a longer duration of the operation as well as imperfect aesthetic outcomes, including the presence of contour deformities due to the robustness of free flaps [2]. In our experience, most of the patients in our cohort had a significant recurrence rate of aggressive, anaplastic RIMs, ranging from 1 to 8 recurrent meningiomas per patient (Figure 6), or were contraindicated for prolonged times in general anesthesia.

**Figure 7:**
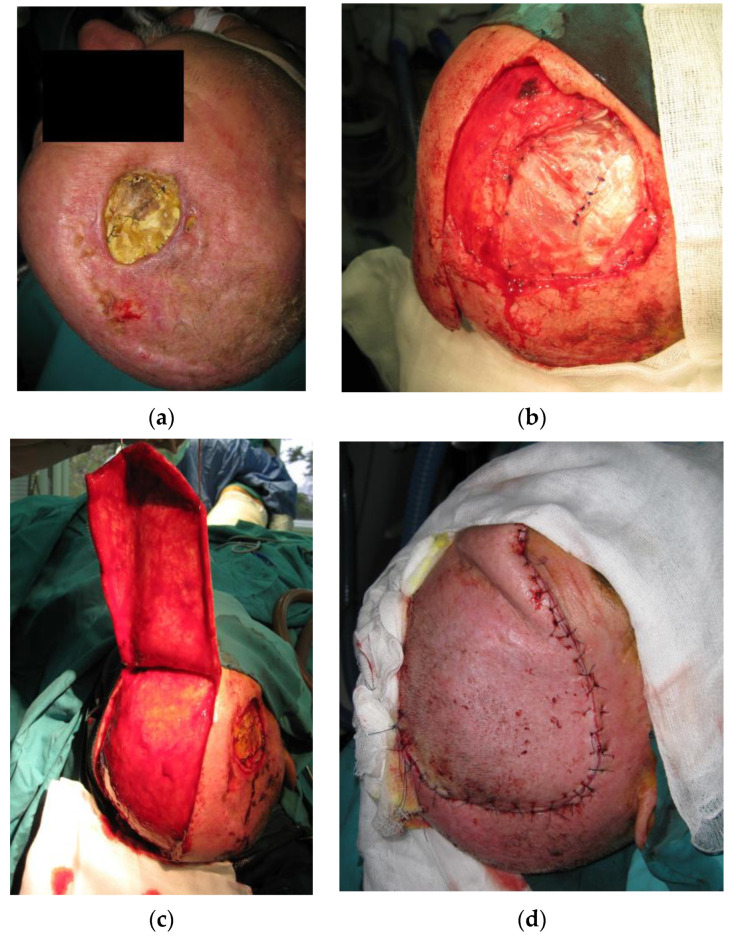
(**a**) Patient with a history of 3 previous RIMs presenting with tissue necrosis and exposure of artificial dura replacement 5 months after adjuvant radiotherapy. (**b**) After excision of the atrophic and unviable tissue, the removal of the exposed artificial material followed. Dura reconstruction was performed using fascia latta. (**c**) The defect was reconstructed with a transpositional flap with the flap donor site reconstruction by autologous skin grafts. (**d**) The flap survived without complications.

Combined with advanced age and comorbidities, the use of microvascular flaps was often contraindicated. When a significant amount of vascularized tissue was required, the use of local or regional flaps was eventually efficiently utilized (Figure 7). As expected, in the case of the regional flap, the reconstruction resulted in the disturbance of the hair pattern as well as contour deformities due to the volume of the flap. Nonetheless, the surgery obtained optimal results given the challenges. The use of microvascular free flaps should be considered sparingly taking into consideration the aggressive nature of the tumor and the probability of recurrence, as well as the patient’s comorbidities, in which case such surgery could be considered a waste of free flap resources for reconstruction.

Additional factors taken into consideration when choosing the adequate reconstruction method is the need for adjuvant radiotherapy. If radiotherapy after tumor resection is indicated, flap coverage is the only adequate choice. When designing the flap, regional or free flaps are a good choice, while local flaps should be taken into consideration only if the donor site is able to be closed primarily [32]. Skin grafts’ tolerance of radiation therapy has been documented and described in practice as well as in the literature, but only in cases when skin grafts were placed on a well-vascularized bed [33]. Patients irradiated for tinea capitis in childhood do not match these criteria. The use of skin grafts for the tumor excision or the flap donor site in this particular situation is not advised.

**Figure 8:**
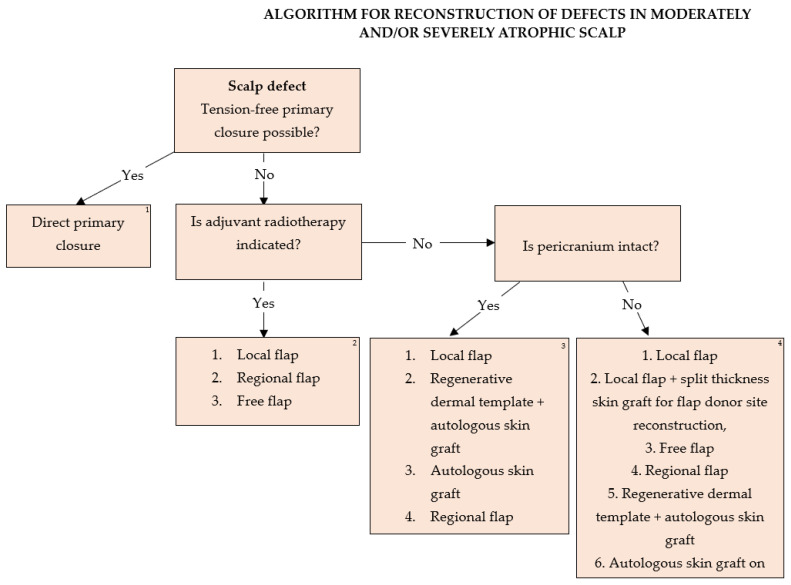
Algorithm for reconstruction of defects in moderately and/or severely atrophic scalp. ^1.^ Direct primary suture is not influenced by decisions on adjuvant radiotherapy or underlying pathology if radicality of primary tumor excision is attainable. ^2.^ All tumors with an indication for adjuvant radiotherapy should be reconstructed with flaps. Flap type should be decided based on defect localization, size, depth, availability of healthy donor tissue, and patient comorbidities in terms of general anesthesia duration necessary for surgery performance, as well as tumor aggressiveness and the possibility of tumor recurrence. The use of autologous skin grafts for primary defect or local flap donor site reconstruction in these patients is generally not advised due to radiation-induced vascular impairment of the scalp tissue. Galeal scoring of the flaps as a means of increasing flap length is not advised in these patients as it presents additional trauma to the radiation-damaged skin and impairs flap integrity. ^3.^ The reconstructive lift in case of intact pericranium in patients without the need for further radiation therapy is based on the accessibility of healthy donor tissue, chance of tumor recurrence, and factors attributing to general anesthesia duration and contraindications. Autologous skin grafts should be used as a last resort in the event of post-excisional defects due to aggressive, high-risk, recurrent tumors. Dermal regeneration templates covered by autologous skin grafts have both an aesthetical and functional advantage due to the thicker dermal component and better resistance to trauma compared to skin grafts, though limited by their cost. Full-thickness skin grafts obtain preferable cosmetic results compared to split thickness skin grafts, but are of limited size and availability. Sheet grafts should be given an advantage compared to meshed grafts for optimal cosmetic results. Free flaps should be taken into consideration sparingly in the event of high tumor recurrence and a need for further multiple surgeries with larger defect reconstruction. ^4.^ In the event of full-thickness scalp defects affecting all structures, reconstruction of dura and the calvaria bones in some cases is necessary. Fascia latta and neuropatch present good choices for dura reconstruction, while implantation materials such as titanium mesh, MMA, the combination of the two, or bone transplants can be used for bone reconstruction. All implantation material should be covered with flaps. Dead space can be efficiently closed with fat grafts. Sparing surgeries involving local flaps should be given an advantage over free flaps, with the donor sites reconstructed with split-thickness skin grafts if further radiation therapy is not indicated. Dermal regeneration templates covered by autologous skin grafts provide sufficient tissue support while reducing surgery duration. Direct coverage of dura or bone spongiosis with skin autografts should be a last-resort option.

Based on our experience, an algorithm was developed for the treatment of moderately or severely atrophic scalp skin such as in tinea capitis patients (Figure 8).

The algorithm could be found useful for the reconstruction of scalp defects in patients after receiving radiation therapy for any reason if severe complications of the treatment occur in the setting of severe skin atrophy with limited reconstruction resources.

**Figure 9:**
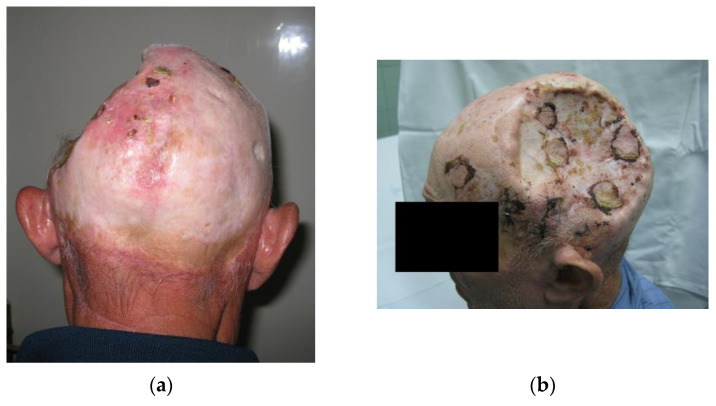
A 67-year-old miner, with lung silicosis, chronic obstructive pulmonary disease, and a history of myocardial infarction was operated on due to aggressive, recurrent skin tumors in 10 previous attempts. Ultrasonographic examination showed poor vascular support of the temporal artery as a recipient vessel for a microsurgical procedure. Recipient blood vessels of the neck were evaluated but the patient was found ineligible for such a procedure due to multiple comorbidities. Eventually, an autologous skin graft was successfully placed directly on dura once all other options were exploited. (**a**) Posteroanterior view; (**b**) Lateral view.

Additionally, the use of dermal substitutes such as Integra^®^, covered by skin autografts could be a good choice in irradiated patients [20,34,35]. This method reduces the perioperative risks associated with the duration of general anesthesia, and the morbidity of the donor sites and could be recommended for the reconstruction of full-thickness scalp defects, especially in patients after several previous surgeries with other reconstructive options exhausted, or with unreliable vascular supply [34,35]. Although some authors recommend this approach as the first line of treatment, the high price of dermal substitutes remains a barrier to its widespread use [34]. Additionally, the application of dermal substitutes is a two-step process, requiring multiple surgeries which should be taken into consideration (Figure 9).

### Limitations

This study is limited by its retrospective nature and the biases associated with such a study design, as well as a small population sample. Larger studies are necessary for the improvement of the algorithm. Sharing surgical experience in such an unusual setting is of utmost importance. Similarly, the nature of the exact data on defect size prior to reconstruction is an important limitation, as these data were obtained retrospectively, from photographs and medical files. Another important limitation of this study is the absence of exact tinea capitis treatment protocols. Such information would significantly improve the understanding of the correlation between radiation doses and tumor occurrence, recurrence, and malignant potential, as well as the dose-dependent consequences of skin atrophy. Additionally, the exact information on the patient’s age at the time of radiation could reveal better-quality information on the latency period.

## 5. Conclusions

Reconstruction of the irradiated scalp remains a challenge in reconstructive surgery. Unfortunately, many protocols developed for scalp reconstruction are not applicable in the setting of severe or diffuse scalp skin atrophy associated with high tumor recurrence rate and radiation-induced vascular impairment, such as in tinea capitis patients in Serbia. An algorithm has been developed based on the authors’ experience in managing these patients. The algorithm could be found useful for the reconstruction of scalp defects in patients after receiving radiation therapy due to any tumor if severe complications of the treatment occur in the setting of severe skin atrophy with limited reconstruction resources.

## Figures and Tables

**Table 1:** Characteristics of study population.

Variables	*n* = 37
Male, *n* (%)	24 (64.9)
Female, *n* (%)	13 (35.1)
Age, mean ± sd	60.6 ± 7.8
Skin tumor, *n* (%)	32 (86.5)
Skin tumor recurrence, *n* (%)	16 (43.2)
BCC, *n* (%)	31 (83.8)
SCC, *n* (%)	7 (18.9)
Meningioma, *n* (%)	16 (43.2)
Meningioma recurrence, *n* (%)	7 (18.9)
Multiple skin lesions at presentation, *n* (%)	24 (64.9)
Aseptic bone necrosis, *n* (%)	7 (18.9)
Thyroid cancer, *n* (%)	3 (8.1)
Comorbidities, *n* (%)	25 (67.6)
Cardiovascular comorbidities, *n* (%)	18 (48.6)
Pulmonary comorbidities, *n* (%)	4 (10.8)
Diabetes Mellitus, *n* (%)	7 (18.9)
Other comorbidities, *n* (%)	5 (13.5)

**Table 2:** Characteristics of patients presenting with aseptic necrosis and association with the underlying defect pathology, reconstructive method, and complications.

	Aseptic Bone Necrosis	
	No *n* = 30	Yes *n* = 7
Gender, *n* (%)		
Male	18 (60.0)	6 (85.7)
Female	12 (40.0)	1 (14.3)
Age, mean ± sd	61.5 ± 7.5	56.4 ± 8.0
Skin tumor, *n* (%)	25 (83.3)	7 (100)
Skin tumor recurrence, *n* (%)	10 (33.3)	6 (85.7)
BCC, *n* (%)	24 (80.0)	7 (100)
SCC, *n* (%)	6 (20.0)	1 (14.3)
Meningioma, *n* (%)	15 (50.0)	1 (14.3)
Meningioma recurrence, *n* (%)	6 (20.0)	1 (14.3)
Multiple skin lesions, *n* (%)	18 (60.0)	6 (85.7)
Thyroid cancer, *n* (%)	3 (10.0)	0 (0)
Primary suture, *n* (%)	9 (30.0)	1 (14.3)
Autologous skin autograft, *n* (%)	14 (46.7)	6 (85.7)
Local flap, *n* (%)	27 (90.0)	7 (100)
Complications, *n* (%)	9 (30.0)	7 (100)
Flap necrosis, *n* (%)	7 (23.3)	3 (42.9)
Partial skin graft loss, *n* (%)	1 (3.3)	5 (71.4)
Infection, *n* (%)	1 (3.3)	3 (42.9)
Dehiscence, *n* (%)	1 (3.3)	1 (14.3)
Comorbidities, *n* (%)	18 (60.0)	7 (100)

**Table 3:** Distribution and severity of skin atrophy.

Skin Atrophy	Frontal	Occipital	Temporal	Parietal
Moderate, n_r_ (%)	7 (18.2)	9 (23.68)	14 (36.84)	16 (42.11)
Severe, n_r_ (%)	3 (10.5)	6 (15.8)	8 (21)	12 (31.58)
Total, n_p_ (%)	27 (72.97)			

n_r_—number of regions, n_p_—number of patients.

## Data Availability

Not applicable.
